# Generation and maintenance of kidney and kidney cancer organoids from patient-derived material for drug development and precision oncology

**DOI:** 10.1016/j.omtm.2024.101368

**Published:** 2024-11-05

**Authors:** Jakub Gubala, Valentin Mieville, Daniel Benamran, Jean-Christophe Tille, Massimo Valerio, Patrycja Nowak-Sliwinska

**Affiliations:** 1 Molecular Pharmacology Group, School of Pharmaceutical Sciences, University of Geneva, 1211 Geneva, Switzerland; 2 Institute of Pharmaceutical Sciences of Western Switzerland, University of Geneva, 1211 Geneva, Switzerland; 3 Translational Research Center in Oncohaematology, 1211 Geneva, Switzerland; 4 Division of Urology, Geneva University Hospitals, Geneva, Switzerland; 5 Division of Pathology, Geneva University Hospitals, Geneva, Switzerland

**Keywords:** patient-derived organoids, renal cell carcinoma, kidney, precision oncology, toxicology, isolation procedure, drug screening

## Abstract

Despite significant advancements in targeted- and immunotherapies, millions of patients with cancer still succumb to the disease each year. In renal cell carcinoma, up to 25% of metastatic patients do not respond to first-line therapies. This reality underscores the urgent need for innovative or repurposed therapies to effectively treat these patients. Patient-derived organoids represent a promising model for evaluating treatment efficacy and toxicity, offering a potential breakthrough in personalized medicine. However, utilizing organoid models for drug screening presents several challenges. Our protocol aims to address these obstacles by outlining a practical approach to successfully isolate and cultivate patient-derived renal cell carcinoma and kidney organoids for treatment screening purposes.

## Graphical abstract

**Figure:**
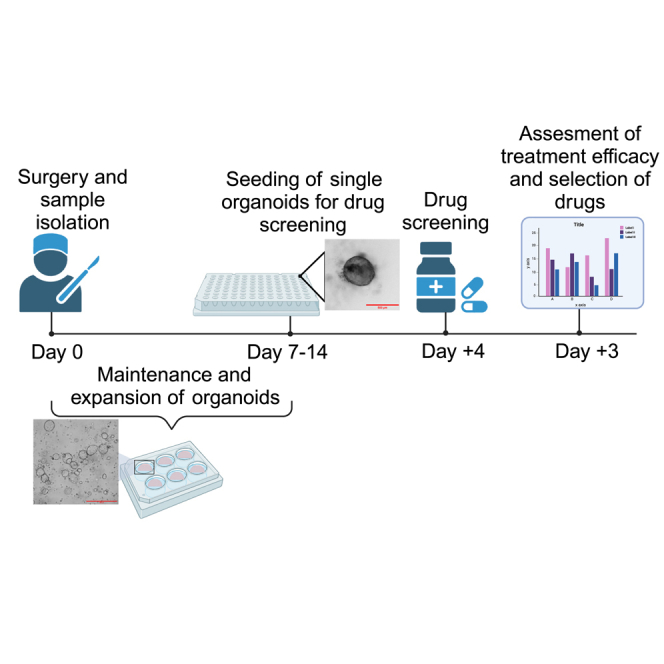

## Teaser

Patient-derived organoids represent a promising model for evaluating treatment efficacy and toxicity, offering a potential breakthrough in personalized medicine. Nowak-Sliwinska and colleagues present a detailed protocol for obtaining kidney and kidney cancer organoids for drug development and precision oncology.

## Introduction

Every year, millions of people worldwide die from cancer.^1^ Advances in the development of more effective treatment options, such as targeted therapies and immunotherapies, have helped increase the overall survival rates.^2^^,^^3^ Nevertheless, there is still a major gap in the field, as not all patients can benefit from existing therapies.^4^^,^^5^ This is notably true for kidney cancer and, more precisely, renal cell carcinoma (RCC). Kidney cancer accounts for around 430,000 cases of cancer worldwide, with around 180,000 related deaths.^6^ RCC accounts for around 90% of all kidney cancer cases.^7^^,^^8^^,^^9^ Its 5-year overall survival is estimated at 78%, with a drop to 15% for stage 4. In addition, it is estimated that 10%–25% of patients with metastatic RCC do not respond to the first-line therapy.^10^ This highlights an unmet need for the development of new treatments and the use of a more personalized approach in the use of existing treatments.

Bridging this gap has become more and more difficult, as developing new therapies is getting increasingly costly,^11^^,^^12^ and an astonishing proportion of lead drug candidates fail to reach the market.^12^^,^^13^ Although generally accepted, the use of cell lines in two-dimensional (2D) cultures is known to be a suboptimal platform due to the lack of the third dimension and the cellular crosstalk.^14^^,^^15^^,^^16^ Consequently, while effective for high-throughput screening, this model often fails to recapitulate most features of organs and tumors and impairs further translation to more complex models.^17^ Spheroids are a model higher in complexity, as they add another dimension allowing for cell-cell interactions and possess additional unique features such as the gradient of oxygen or nutrients.^18^ They were already shown as a better model for drug screening in comparison to 2D culture, as the crosstalk can influence the treatment response.^19^^,^^20^ However, spheroids are often comprised of established cell lines that might not respond to treatment as patient cells would.^21^ This discrepancy between a model used for screening and the reality of the clinics is not negligible when considering the observed differences in efficacy and toxicity between the outcomes of most *in vitro* experiments and future translation into clinical trials.^13^ Thus, we hypothesize that the use of a model closer to the reality of the patient, such as organoids, can help bridge the gap by providing more accurate insights into drug responses and push us closer to personalized treatments.^22^^,^^23^^,^^24^^,^^25^^,^^26^

Organoids are complex 3D *in vitro* structures that can mimic the function, structure, and heterogeneity of human tissues.^27^^,^^28^^,^^29^^,^^30^^,^^31^^,^^32^^,^^33^ They are comprised of either induced pluripotent stem cells (iPSCs), embryonic stem cells (ESCs), adult stem cells, or tumor cells. These cells not only proliferate but also can self-organize and differentiate into these complex organ-like structures.^22^^,^^34^^,^^35^ Organoids have already been shown to exhibit a high degree of similarity to corresponding biological tissue in morphology and function.^34^^,^^36^ In addition, organoids allow for the study of toxicity and disease mechanisms, as well as for the detection of potential targets, that, in a 2D setting, would be missed. As such, they are expected to better emulate the patient’s response to a treatment.^37^

The organoid model, however, retains some limitations.^38^^,^^39^ Differentiation of non-cancerous organoids from iPSCs or ESCs is a long process, and the end product can include undesired cell types because of unspecific differentiation.^40^ There can also be problems of scalability, as not all methods provide options for high-throughput experiments such as drug screening. Organoid culture allowing for such experiments can require costly and/or self-manufactured materials such as scaffold plates or liquid handling robots.^41^^,^^42^^,^^43^ The study of cancer using iPSC- or ESC-derived organoids is also possible by introducing mutations in non-cancerous organoids or through reprogramming cancer cells into iPSCs. Reprogrammed patient cancer cells could be used to estimate the patient response to therapy but would require re-differentiation. Hence, iPSC- or ESC-derived organoids are typically used to study mechanisms such as cancer initiation and progression.^44^

**Figure 1:**
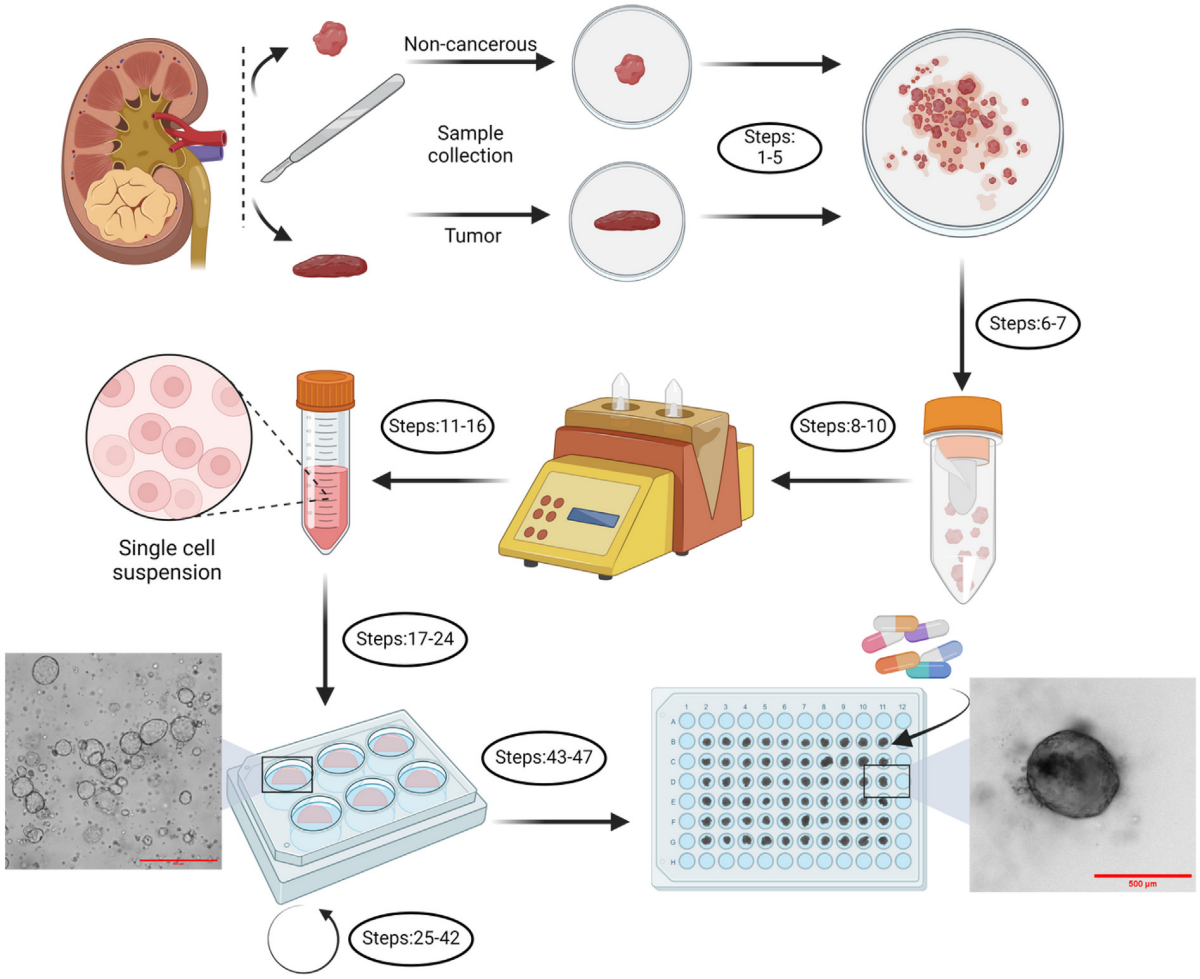
Graphical representation of the protocol Steps 1–5: mechanical fragmentation, steps 6–10: enzymatic digestion, steps 11–24: washing and plating of the cells, steps 25–42: passaging and maintenance of the organoids, and steps 43–47: seeding of single organoids for use in drug screening. Created with Biorender.com.

Unlike ESC and iPSC organoids, adult stem cells and cancer cell-derived organoids are directly isolated from the tumor or organs and already display patient-specific characteristics. As such, these patient-derived organoids (PDOs) do not necessitate complex differentiation or gene editing procedures. Thus, PDOs provide a simpler way to assess the response of patients with cancer to therapy.^44^ Here, we present a detailed and annotated protocol (Figure 1) to isolate and cultivate PDOs isolated from RCC and adjacent kidney tissue. This protocol was based on the comparison of research articles using similar 3D models of RCC^45^^,^^46^^,^^47^^,^^48^ with existing protocols developed by our group for other types of tumors and tissues. The protocol was further adjusted throughout our 3 years of experience using this model. Adjustments were focused on improving practicality and troubleshooting encountered difficulties. Moreover, we also provide an accessible way of using these organoids to evaluate drug sensitivity in an efficient manner.

## Required materials

### Reagents and equipment

All reagents and equipment used are listed in Table 1.

**Table 1:** List of all equipment and reagents/solutions used in the protocol

Name	Company	Catalog number
**Equipment**		

96-well plate black side/clear flat bottom	Corning	3651
cell culture 6-well plate	Greiner	7.657 160
cell strainer 100 um	Greiner	7.542 000
Cytation 3/5	BioTek	CYT3MFV/CYT5
FlashFREEZE	Milestone Medical	N/A
GentleMACS C Tubes	Miltenyi	130-093-237
GentleMACS Octo Dissociator with Heaters	Miltenyi	130-096-427
glass Petri dish	Brand	BR455701-10EA
hemacytometer - Neubauer chamber	Marienfield	640110
microplate, 96-well plate, PS, U-bottom	Greiner	650970
Peel-A-Way embedding molds	Sigma	E6032-1CS
serological pipette 10 mL	Sarstedt	86.1254.001
serological pipette 25 mL	Corning	4489-COR
stainless-steel forceps	Bochem	10464881
surgical blade	Paramount	132521
T25 culture flask	TPP	9026
tips, p1000	Clearline	713112
tips, p200	Dutscher	862527A
tubes, 50 mL	Greiner	7.227 261
tubes, 2 mL	Axygen	MCT-200-C

**Reagent/solutions**		

ACK lysing buffer	Gibco	A1049201
CellTiter-Glo 3D Cell Viability Assay	Promega	G9681
Collagen I, rat tail^a^	Gibco	A1048301
Corning Cell Recovery Solution	Corning	354253
Corning Matrigel Basement Membrane Matrix^b^	Corning	354234
DMEM/F-12, GlutaMAX supplement	Gibco	10565018
DMSO	Sigma-Aldrich	41640
EasySep Dead Cell Removal (Annexin V) kit	STEMCELL Technologies	17899
fetal bovine serum (FBS)	Biowest	S1810-500
HBSS, w/o calcium and magnesium	Gibco	14170088
human basic fibroblast growth factor/FGF2	R&D Systems	3718-FB
human epidermal growth factor	Gibco	AF-100-15
human Noggin, research grade	Miltenyi	130-103-456
Liberase DH research grade	Roche	05401054001
OCT mounting media	VWR	361603E
PBS	Gibco	10010015
penicillin-streptomycin	Amimed	4-01F00-H
platelet-derived growth factor-BB human	Sigma-Aldrich	P3201
primocin	Invivogen	ant-pm-1
recombinant human FGF-10 protein	R&D Systems	345-FG-025
recombinant human R-Spondin 1 protein	R&D Systems	4645-RS-025
recombinant human R-Spondin 3 protein	R&D Systems	3500-RS-025
recombinant human Wnt-3a protein	R&D Systems	5036-WN-010
Renal Epithelial Cell Growth Medium 2	Promocell	C-26130
StemPro hESC	Gibco	A1000701
Trypan blue solution, 0.4%	Gibco	15250061
TrypLE Express enzyme (1×)	Gibco	12604013

a Protocol established using collagen I. Use of another collagen may affect the growth of the primary cells.

b Protocol established using Matrigel. Substitutions may affect the growth of organoids.

### Reagent setup

Before each isolation, it is crucial to prepare all reagents needed for the procedure, as all tasks need to be completed as soon as possible in order to limit cell death and loss of sample.

#### Media and cell culture conditions

• Transport medium: DMEM/F12 + GlutaMAX medium supplemented with 1× Primocin
• Wash solution: HBSS without (w/o) calcium and magnesium
• Digestion medium: DMEM/F-12 + GlutaMAX supplement medium with 5 μg/mL Liberase DH
• Digestion arrest solution: RPMI or DMEM + 10% FBS
• Freezing medium: FBS + 10% DMSO

Note: to be prepared directly before use.

• Culture medium: isolated cells that are cultivated in 3D are kept in
  ○ Cancer cells: DMEM/F12 + GlutaMAX medium supplemented with 0.02 μg/mL human epidermal growth factor (hEGF), 0.001 μg/mL human basic fibroblast growth factor (hFGF), 0.02 μg/mL fibroblast growth factor 10 (FGF10), 0.02 μg/mL R-Spondin 1, 0.01 μg/mL R-Spondin 3, 0.1 μg/mL human Noggin, 2.5 μg/mL Wnt3a, 0.005 μg/mL platelet-derived growth factor BB, 1× Primocin, and 1× StemPro. The role of each component in the medium can be found in Table S1.
  ○ Cancer and non-cancerous cells: complete Renal Epithelial Cell Growth Medium 2 with the addition of 1% P/S.

Critical step: it is crucial to store the medium at 4°C for no more than 2 weeks prior to use and to avoid repeating cycles of warming up and cooling down.

Isolated cells cultivated in a 2D monolayer are kept in complete Renal Epithelial Cell Growth Medium 2 with the addition of 1% P/S in a flask coated with rat tail collagen I.

### Equipment setup

Critical step: be prepared before beginning the isolation procedure.

I) Pre-warm the 6-well plate.
  (a) Place the plate in the incubator at 37°C, 5% CO_2_, a minimum of 2 h before the intended use, preferably overnight.
II) Pre-chill all solutions and pieces of equipment prior to contact with Matrigel.
  (a) Place the sterile pipette tips P200 and P1000 at −20 °C at least the day before expected isolation and keep until just before the intended use.
III) ∗(OPTIONAL) Prepare coated T25 flasks.
  (a) Coat a T25 flask with rat tail collagen I (diluted in cold PBS) at 50 μg/mL, 100 μL/cm^2^ of the plate.
  (b) Incubate the flask at 37°C, 5% CO_2_, for a minimum of 2 h, preferably overnight.
  (c) Discard the collagen solution and wash the flask with PBS just before seeding the cells.
  (d)Note: any other 2D culture dish can be used accordingly.

### Patient material transportation conditions

Cancerous and (upon availability) non-cancerous kidney material is retrieved just after surgical resection. Each sample is placed in a different 50 mL tube half-filled with transport medium and kept on ice until the start of the isolation procedure. Processing of the sample is performed in the following hours.

### Necessary allowances and collaboration establishment

All patient-derived material was obtained via the protocol approved by Swiss ethics committees on research involving humans (2017-00364) upon signed patient’s consent.

### Safety precautions, procedures, and conditions before sample recuperation

All patient-derived material must be coded to ensure the privacy of the patient. A coded list of patients (2017-00364 …) including information on respective RCC (PRCC …) and non-cancerous (PHK …) can be found in Table S2.

All materials of human origin might contain viruses and other agents. Samples should be handled with extreme care, as they pose a risk of infection if not handled properly. Samples must be processed in biosafety level 2 (BSL-2) facilities by trained staff in authorized laboratories. In our study, we exclude samples where the patient tested positive for HIV-1, -2, HTLV-1, or hepatitis B or C.

## Procedure

As a matter of clarity, the following listed procedure will assume the availability of a single sample. However, multiple cancerous and non-cancerous samples can be processed in parallel following the same steps.

### Mechanical fragmentation

(1) Wash samples 1× with 20 mL HBSS and mix by inverting the tube.
(2) Transfer the sample to a glass Petri dish using dissecting forceps. At this point, any black necrotic areas are removed using surgical blades. Surgical blades are also used to isolate small fragments (recommended size: 1–10 mm^3^, depending on the availability of tissue) of the patient samples to be cryopreserved in optimal cutting temperature (OCT) mounting medium and, thereafter, conserved at −80°C using FlashFREEZE.
(3) Remaining biological tissues are transferred into 2 mL tubes to be weighted on an analytical scale.
(4) Transfer back the sample onto the Petri dish and add 1 mL of the digestion medium.
(5) Use two surgical blades to cut the tissue into small fragments of ∼1 mm^3^.

### Enzymatic digestion

(6) Transfer the content of the Petri dish into a GentleMACS tube.
(7) Add 9 mL of digestion medium in the tube containing the sample. Use those 9 mL to thoroughly wash the Petri dish to lose a minimum of sample in the transfer process.
(8) Incubate for 1 h at 37°C under agitation in a GentleMACS tissue dissociator using the embedded 37C_h_TDK_1 program.
(9) Strain the content of the tube through a 100 μm strainer inserted in a new 50 mL tube. Rinse the GentleMACS tube with 10 mL of medium containing 10% of FBS and strain content in the same 50 mL tube to stop the enzymatic reaction in the flowthrough (FT).
(10) Use 20 mL of the same FBS-containing serum to collect the retained fraction (RF) from the strainer and transfer it to another 50 mL tube.

### Plating of the cells

**Figure 2:**
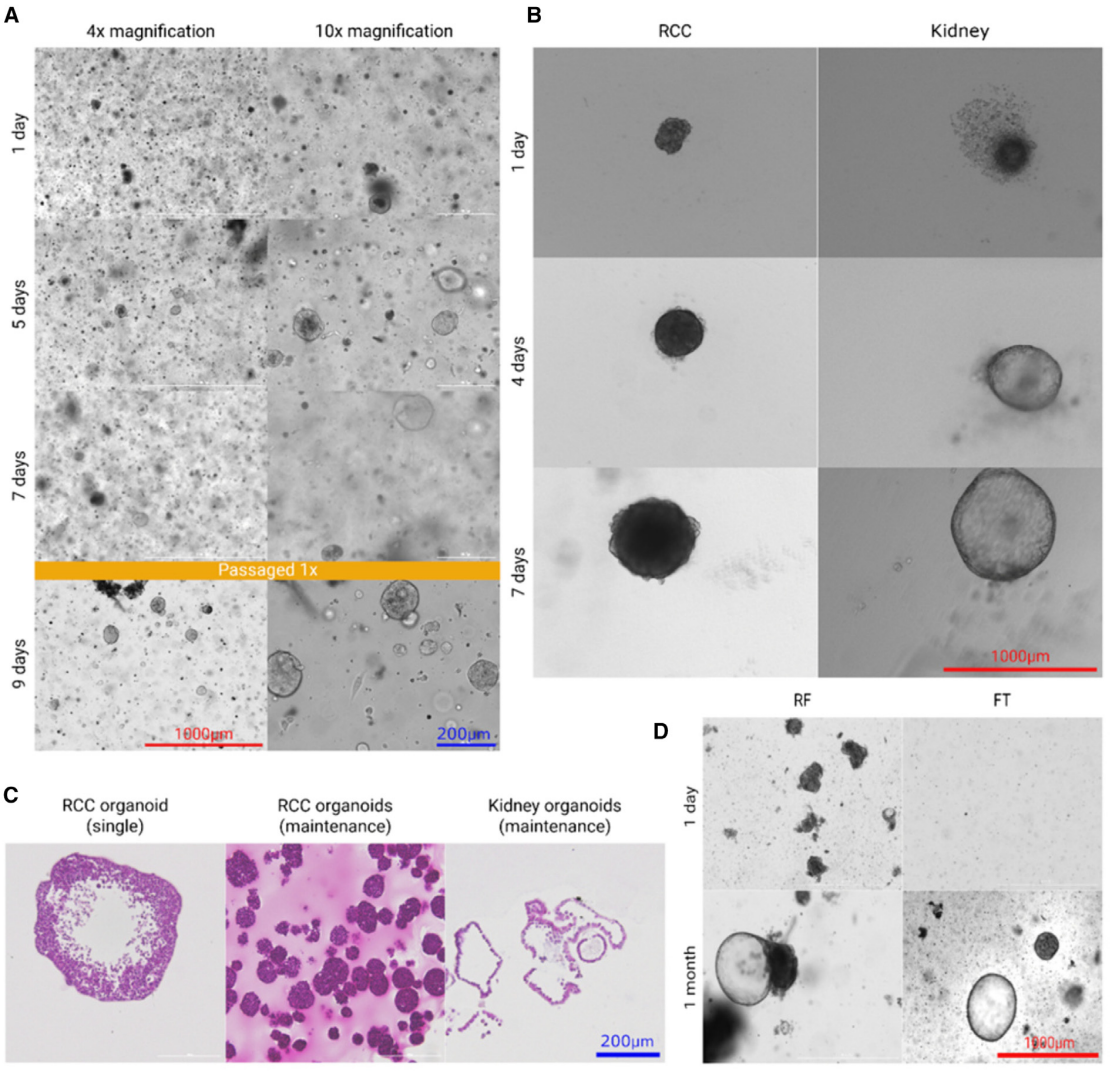
Organoid kinetics (A) Formation of organoids freshly isolated from the cancer sample PRCC19 as observed in the maintenance plate. Imaging was performed on days 1, 5, 7, and 9 after isolation using Cytation3 in brightfield with 4× magnification (left column) and 10× magnification (right column). (B) Representative pictures of single organoid formation in a 96-well plate. Imaging was performed using Cytation3 in brightfield with 4× magnification on days 1, 4, and 7 after seeding of PRCC09 (left column) and kidney sample PHK22 (right column). (C) Hematoxylin and eosin staining of 5 μm sections of organoids (from left to right): PRCC09 as single organoid, PRCC09 as in a maintenance plate, and PHK18 as in a maintenance plate. Imaging was performed at 20× magnification using Cytation5 in color bright-field mode. (D) Formation of kidney organoids isolated from the retained fraction (left) and flowthrough (right) of patient PHK18. Imaging was performed using Cytation3 in brightfield with 4× magnification. Red and blue scale bars represent 1,000 and 200 μm, respectively, in the column they appear.

(11) Centrifuge both tubes at 300 *g* for 5 min, then remove the supernatant of both tubes.
(12) Resuspend the FT with 10 mL ACK lysing buffer and incubate for 5 min at room temperature.
(13) Centrifuge at 300 *g* for 5 min, then remove the supernatant.
(14) Wash both RF and FT with 20 mL HBSS.
(15) Centrifuge at 300 *g* for 5 min, then remove the supernatant.
(16) Resuspend the RF in 2 mL of culture medium and directly transfer it to a 6-well plate well.
  (a)Note: the amount of medium and number of wells might need to be increased depending on the volume of the RF.
(17) Count the cells of the FT.
(18) Mix the FT in 1:1 ratio with Matrigel in an Eppendorf held on ice.
  (a)Note: higher concentrations of Matrigel do not seem to provide any benefit to the isolated cells. However, at lower Matrigel concentrations, a higher number of cells attach to the bottom of the culture plate.
  (b)Critical: once the cells are mixed with Matrigel, work as quickly as possible in order to minimize the chances of the Matrigel solidifying in the tube or pipette tip.
(19) Distribute 100–200 μL drops in each well of a 6-well plate.
  (a)Critical: avoid introducing bubbles into the Matrigel domes, as they will interfere with the imaging of organoids and can cause uneven distribution of organoids.
  (b)Note: 24-well plates can be used with smaller drops of cells in Matrigel (40 μL). However, we found those to be harder to retrieve during the passaging procedure, resulting in a higher loss of material.
  (c)Note: multiple drops of Matrigel can be placed in the same well to limit the use of culture medium.
  (d) ∗OPTIONAL: seed 50,000–100,000 cells in a T25 flask for 2D culture. Use the Renal Epithelial Cell Growth Medium 2. 2D cells can be later retrieved and seeded into single spheroids. Splitting of the cells should be done according to the standard 2D culture splitting protocol before cells reach 100% confluency.
  (e)Note: it is possible to retrieve cells plated in 2D and plate them in either a maintenance plate or 96-well plate as spheroids. We observed in the case of cancer cells that some will still grow in 3D when transferred into a maintenance plate at an early passage. Keeping cells in 2D is merely an insurance to increase the chances of expanding the patient material in case it fails to produce organoids in the Matrigel domes and is by no means the preferred option.
(20) Incubate plates with Matrigel for 30 min at 37°C and 5% CO_2_.
  (a)Critical: in the case of shorter incubation, Matrigel may dissolve when adding culture medium.
(21) Once the Matrigel domes have solidified, gently add culture medium to the well until the domes are fully submerged.
  (a)Critical: medium must not be added directly on top of the Matrigel domes, as doing so might disrupt them.
  (b)Note: for the tumor samples, we primarily use culture medium A mentioned above; however, we noticed that some cancerous samples grow better in culture medium B. We therefore keep part of the sample in both media. For non-cancerous samples, use culture medium B.
(22) Incubate at 37°C and 5% CO_2_
(23) Replace the medium every 5–7 days.
  (a)Note: example of organoid growth can be observed in Figures 2A, 2B, and 2D.
(24) Passage when organoids are 300–500 μm or when the medium turns yellow.
  (a)Critical: ensure the organoids are not split too quickly, as doing so might lead to termination of the culture.
  (b)Critical: ensure optimal seeding density for the first few passages.
  (c)Note: it is sometimes possible for the Matrigel dome to detach from the bottom of the plate and freely float in the medium. In this case, it is recommended to pass the organoids, even at a 1:1 ratio, to ensure proper growth.
  (d)Note: medium that turned yellow can be replaced by a fresh one. However, from our experience, fresh medium will again turn yellow in the next 24 h. Changing the medium daily can be both costly and time consuming; thus, we strongly suggest passaging the organoids.

### Passaging and single organoids seeding protocol

Critical: before beginning, pre-warm the culture medium in the water bath (for the maintenance plate) and the 6-well plate in the incubator at 37°C. The plate should be pre-heated ideally overnight or at least 2 h before intended use.

Critical: all material coming into contact with unpolymerized Matrigel (pipette tips, reservoirs, U-bottom shape 96-well plate) is kept at −20°C and taken out of the freezer right before use and should be placed at −20°C at least 1 day before intended use.

(25)Gently remove the medium from the well without breaking the Matrigel dome.
  (a)Note: if parts of the domes detached, collect all the liquid in a 50 mL Falcon and spin for 5 min at 300*g* to separate the liquid from the cells.
(26) Hold the plate at a 45° angle and gently scratch the domes with a cell scraper.
  (a)Note: other protocols usually try to dissolve the dome by pipetting cold Cell Recovery Solution on top of it. While this method works, we found that scraping the dome from the plate leaves fewer cells in the plate while enabling better recovery.
(27) Add 1 mL/plate of cold Cell Recovery Solution on top of where the domes used to be to recover eventual remaining cells.
(28) Pipette the Matrigel domes alongside the Recovery solution and transfer to a 2 mL Eppendorf tube held on ice. Wash the remaining fragments in the well(s) using another 500 μL of Cell Recovery solution.
(29) Pipette up and down in the tube to further dissociate the remanent of the Matrigel domes.
(30) Leave on ice for 20 min with gentle lateral agitation to separate the cells from the Matrigel.
(31) Spin down for 5 min at 300*g* at 4°C, then remove the supernatant.
  (a)Note: if no pellet is visible, spin down again at 4°C, 400*g*, for 5 min.
(32) Add 500 μL TrypLE Express, then incubate for 10 min at 37°C.
  (a)Note: do not hesitate to pipette up and down and vortex multiple times to mix and disrupt a maximum of organoid clumps.
(33) Add 500 μL of FBS and mix well to stop the enzymatic digestion.
(34) Centrifuge 5 min at 300*g*, then remove the supernatant.
(35) Resuspend the pellet in 2 mL of PBS.
(36) Count viable cells using Trypan blue exclusion dye (1:1).
  (a)Note: if only seeding single organoids, skip steps 37–42 and proceed directly to step 43.

### Organoid maintenance

(37) Transfer in a new tube a volume of cell suspension containing 50,000 viable cells per 6-well plate wells.
(38) Complete the tube to half of your final desired volume with cold culture medium. A final volume of 200 μL per 50,000 cells is used to calculate the desired volume.
  (a)Note: if your volume of cell suspension is higher than half of the final volume, spin down for 5 min at 300*g*, then remove as much supernatant as necessary.
(39) Using micropipette tips kept at −20°C, complete your cell suspension to a final volume with Matrigel.
(40) Mix well by pipetting up and down, then transfer your mix as one/multiple dome(s) in a pre-heated 6-well plate.
(41) Incubate 30 min at 37°C, 5% CO_2_, to polymerize the Matrigel.
(42) Cover the dome with pre-heated culture medium and incubate at 37°C, 5% CO_2_.

### Seeding of single organoids

Critical: it is crucial that all these steps are performed on ice, with the material kept at −20°C, and as quickly as possible, as this will directly affect the percentage of single structure formation.

Critical: culture medium, unlike for the maintenance plate, should be used directly from the 4°C storage and, during the procedure, kept on ice.

(43) In a 50 mL tube, dilute the volume necessary to have 1,000 cells per well and *add* the volume of cold culture medium needed to fill the same number of wells up to 80 μL, minus the volume of Matrigel necessary for a final Matrigel concentration of 2.5% (v/v).
(44) Transfer the content of the tube to a cold reservoir and dispense 80 μL per well using a multichannel P200 micropipette with cold tips in a cold low-attachment round-bottom 96-well plate.
  (a)Critical: keep the reservoir and the 96-well plate on ice during the step.
  (b)Critical: avoid using wells on the edges of the plate. Evaporation can introduce variability in your conditions.
(45) Centrifuge the plate at 400*g* for 5 min at 4°C.
  (a)Critical: ensure the cells are centered in the well. In case they are not, centrifuge again once with the same parameters.
(46) Complete empty wells with 80 μL of PBS.
(47) Incubate at 37°C, 5% CO_2_.
  (a)Note: an example of single organoid formation can be found in Figure 2B.

### Cell freezing protocol

(48) Retrieve cells according to passaging and single organoids seeding protocol.
(49) Transfer the cells obtained in step 10 of the passaging and single organoids seeding protocol into a new 15 mL Falcon tube.
(50) Spin the cells for 5 min at 300*g*.
(51) Prepare the freezing container.
  (a)Note: we recommend freezing around 0.5 × 10^6^–1 × 10^6^ cells/cryovial in 1 mL of freezing medium.
(52) Remove the supernatant.
(53) Resuspend the cells in the freezing medium.
(54) Transfer 1 mL into each cryovial.
(55) Close the freezing container and store at −80°C.
(56) The following day, transfer the vials into a liquid nitrogen bank.

### Cell thawing protocol

Critical step: before protocol initiation, pre-warm the medium in the water bath (DMEM + 10% FBS + 1% P/S) and the 6-well plate in the incubator at 37°C. The plate should be pre-heated ideally overnight or at least 2 h before intended use.

Critical step: use chilled pipette tips for any material that has come into contact with liquid Matrigel.

(57) Place the frozen vial in the water bath at 37°C until ice begins to melt.
  (a)Critical step: no more than 2 min.
(58) Disrupt remaining ice by pipetting with a P1000.
(59) Transfer contents of the vial into 50 mL Falcon tube.
(60) While gently shaking the tube, add 9 mL of pre-warmed cell culture medium.
  (a)Critical step: add medium slowly, drop by drop, and mix well via gentle pipetting.
(61) Spin the tube at 300*g* for 5 min.
(62) Remove the supernatant.
(63) Wash the cells with 10 mL of PBS and repeat steps 61–62.
(64) Resuspend the pellet in 1 mL of PBS.
(65) Evaluate viability by counting viable cells using Trypan blue exclusion dye (1:1).
(66) Proceed as described in steps 37–42.

Note: it is recommended to perform a mycoplasma test 1 week after thawing the cells.

### Treatment modalities

Caution: some drugs might be highly cytotoxic, so refer to the safety sheet of each compound. Drugs must be handled only by trained personnel.

(67) 3D structures obtained in 17–22 of passaging and single organoids seeding protocol are left to grow at 37°C, 5% CO_2_, for 96 h after seeding.
(68) All wells are imaged in brightfield using BioTek Cytation3 or -5.
  (a)Note: using z stack acquisition is necessary, as the focus might slightly differ from one plate to another.
  (b)Note: these pictures are used to assess the singleness and homogeneity of the structure(s) in each well. Wells that contain more than one structure or contain fibers are marked as such and will not be used for further experimentation.
    (i)The number of wells excluded for containing multiple structures seems to correspond to the capacity to keep the Matrigel from polymerizing before spinning the plates during seeding. To reduce the number of excluded wells, you might consider decreasing the time that the cell suspension containing Matrigel is exposed to room temperature by reducing the amount of plates seeded in parallel and reduce the temperature of the material in contact with this suspension by taking the plates, reservoir, and pipette tips out of the freezer at the very last second before use.
    (ii)In contrast to the wells containing multiple 3D structures, we do not know whether the presence of fibers can affect the results of a treatment nor their origin. However, we know that they affect the morphology of the 3D structures, as they tend to grow alongside those fibers; thus, we exclude them preemptively.
(69) Drug(s) are conserved in anhydrous DMSO and pre-diluted to 5 times the desired final concentration in cell culture medium.
(70) 20 μL of the drug mix is gently added on top of predefined wells containing 3D structures.
(71) Plates are incubated 72 h at 37°C, 5% CO_2_.
(72) Readout is performed using 3D CTG assay.
  (a)Note: while other assays can and were performed looking for specific outcomes, such as whole-mount staining, histological staining of sections, or flow cytometry analysis of the dissociated structures, 3D CTG is much more suited for higher-throughput treatment screening, as it is easier and quicker to handle and necessitates a lesser amount of sample.
(73) 60 μL of 3D CTG is added to each well using a multichannel P200 micropipette.
(74) Plated are incubated for 30 min in the dark at room temperature.
(75) Content of wells is mixed by gently pipetting up and down 10 times, and 140 μL is then transferred to a clear-bottom black-side 96-well plate. Change tips between every well.
(76) Plates are then read for luminescence emission using Cytation3 or -5.

### Fixation of the organoids

Caution: PFA is highly toxic. Dispose according to local guidelines.

#### Preparation

(77) Coat all material coming into direct contact with organoids with 0.1% BSA in PBS – 1.5 mL Eppendorf tubes and pipette tips. Use P1000 with a cut-off tip.

#### Recovery

(78) Gently recover organoids from the 96-well plate and, in case of the same conditions, pull them together in 1 Eppendorf tube.

#### Washing

(79) Add 1 mL of 0.1% BSA in PBS into each Eppendorf tube.
(80) Allow organoids to sediment for 30 s–1 min.
(81) Remove the supernatant and repeat the wash.

#### Fixation

Caution: to be done under the chemical hood.

(82) Remove as much of the supernatant as possible without disrupting the organoids.
(83) Add 1 mL of PFA to each tube.
(84) Wait about 3 h at room temperature.
  (a)Note: too long of an incubation can lead to loss of antigens, while too short can allow for degradation of the organoids.

#### Washing and storage

(85) Perform 3 washes with PBS at room temperature, allowing organoids to sediment in between the washes.
(86) Fixed organoids can be stored at 4°C.
(87) Fixed organoids can be later used for whole-mount staining or embedded into 2% bacto-agar gel, dehydrated, embedded into paraffin blocks, and sectioned for standard histology staining. An example of such staining can be seen in Figure 2C.

## Timing

Mechanical fragmentation: 25–35 min.

Enzymatic digestion: 1.5–2 h.

Plating of the cells: 1.5–2 h.

Passaging and single organoids seeding protocol: 45 min–1 h.

Organoid maintenance: 45 min–1 h.

Seeding of single organoids: 15–45 min.

Cell freezing protocol: 10–15 min.

Cell thawing protocol: 30–45 min.

Treatment modalities: 1–8 h, depending on the number of conditions tested.

Fixation of the organoids: 3.5–4 h.

## Troubleshooting

Troubleshooting steps and mitigation strategies can be found in Table 2.

**Table 2:** Troubleshooting and mitigation strategies

Step	Problem	Possible cause	Mitigation
41–45	cells do not form organoids after single-cell dissociation	too few epithelial cells were extracted the time between resection from the patient and collection of the sample was too long missing growth factor in the medium it is possible that immune cells present within the sample were targeting cancer in culture	plate cells into 96-well plate (steps 41–45); if in this case, organoids do not form, then the sample will not be usable; for future isolations, ensure the high quality of the sample and respect the recommended weight threshold of the sample ensure that the isolation can commence as soon as the tissue is resected from the patient prepare fresh media containing all growth factors at recommended concentrations perform sorting to deplete immune cells or use any of the available immunosuppressive agents throughout the first few days of culture
20	organoids are not well distributed in the Matrigel and grow on the bottom of the well	Matrigel concentration is lower than 50% domes flattened during transport	after 10–15 min of incubation, flip the plate upside down to ensure more even distribution of organoids pre-heat new well plate before use and wait 3–5 min at room temperature before transferring to the incubator
24c	Matrigel detaches from the plate	the dome did not attach well to the plate the stream of the medium was too strong when immerging the dome the sample contains debris altering the integrity of the gel	pre-heat the new well plate before use use a pipette with a larger opening to reduce applied pressure; direct the flow of medium as far as possible from the Matrigel dome recover the dome, remove Matrigel as when passaging, and wash a couple more times with PBS; if this is not sufficient, then dead cell removal can be performed using any of the sorting strategies—such as EasySep
29	organoids present in Matrigel when retrieving	Cell Recovery Solution was not cold enough to dissolve Matrigel	when splitting multiple plates of organoids at the same time, keep the Cell Recovery solution on ice throughout the entire procedure
40	Matrigel domes fuse when plated in the same well	multiple droplets were too big/too close to each other	use larger/more culture dishes
42	Matrigel dissolved when adding the medium	too short of an incubation or the medium was not pre-heated	allow Matrigel to fully polymerize in the incubator and pre-heat the medium to 37°C for a minimum of 1 h before adding to the plate containing cells
45	cells are not centered in the well	Matrigel polymerized before centrifugation—either before mixing with cells or during the seeding process	keep a few minutes in a cold centrifuge, then perform a second centrifugation; ensure that the temperature in the laboratory stays low all year round and that Matrigel/cell suspension is kept on ice
77–87	loss of organoids	organoids might have been stuck to the pipette tip	ensure the coating of the tip with the BSA solution

## Anticipated results

Our protocol describes a robust method for establishing PDOs from human kidney and RCC tissues. We managed to achieve high efficiency in the establishment of organoids and their expansion—68% for tumor organoids and 84% for healthy kidney organoids (see Table S2).

Organoid formation can be observed between 1 and 14 days after processing of the tissue. Examples of organoid formation can be found in Figures 2A, 2B, and 2D. Non-cancerous samples typically give rise to cystic organoids, whereas in RCC, the morphology is more variable between patients. This might be attributed to the higher heterogeneity of the cancer. RCC organoids might appear cystic or solid, even within the same patient sample. Growth of the organoids is heterogeneous between samples, as some PDOs might require splitting each week at 1:6–1:2, whereas some only once a month at 1:2. In the case of splitting the cells once a month, it is recommended to change the medium every week.

**Figure 3:**
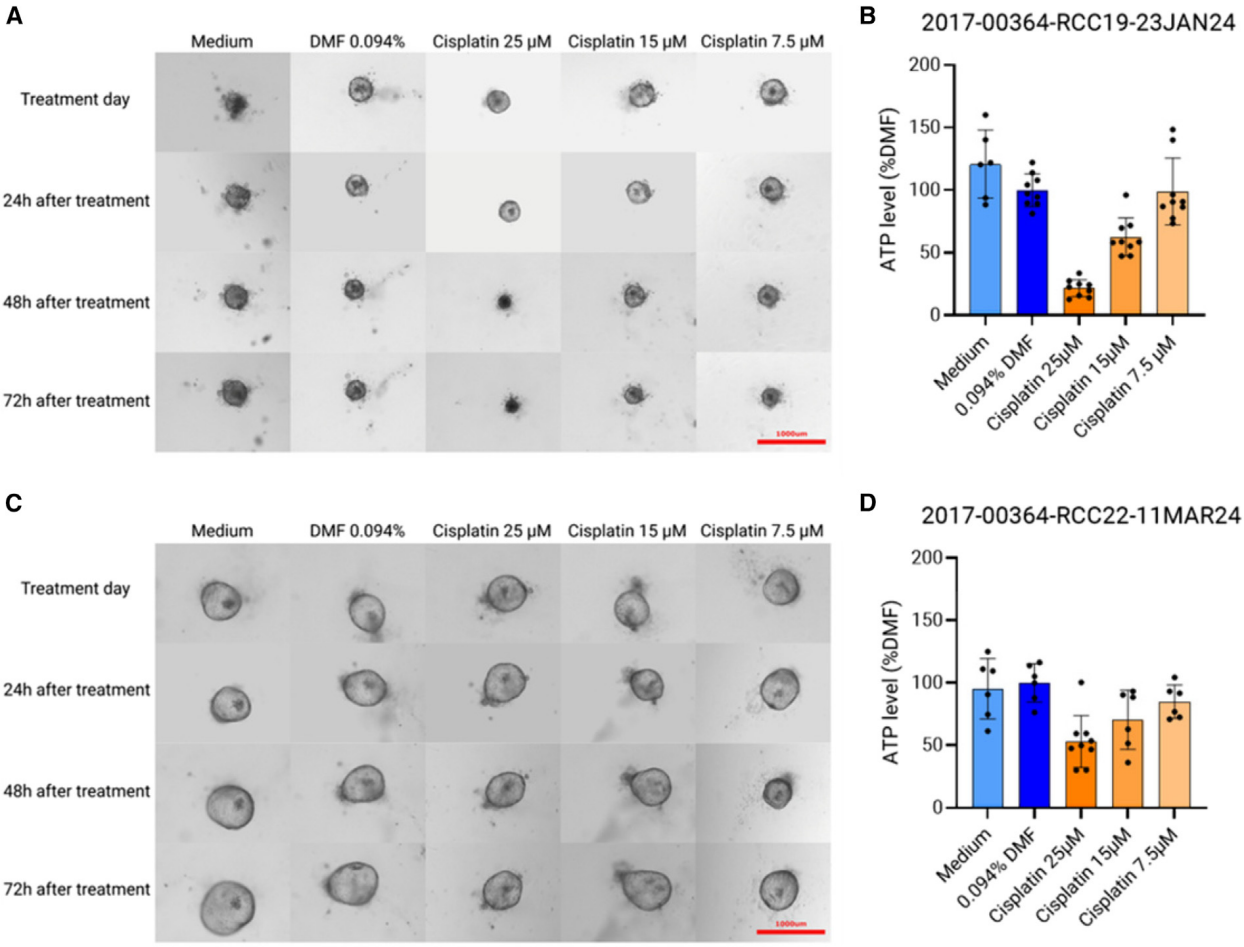
Expected treatment outcome Effect of nephrotoxic cisplatin treatment on kidney organoids cultured from patients, PHK19 (A and B) and PHK22 (C and D). (A and C) Representative pictures of kidney organoids exposed to (from left to right) culture medium, control dimethylformamide (DMF), and 25, 15, or 7.5 μM of the nephrotoxic drug cisplatin. Pictures were taken in brightfield with 4× magnification using Cytation5. Each column displays the same organoid over time (from top to bottom): on the day of the treatment and 24, 48, and 72 h after treatment. Red scale bar represents 1,000 μm and is valid for all pictures. (B and D) Bar graphs showing the ATP level measured in kidney organoids exposed to culture medium (light blue), DMF control (dark blue), and 25, 15, or 7.5 μM of the nephrotoxic drug cisplatin (dark to light shades of orange). All bares are normalized to the 0.094% DMF control, which corresponds to the concentration found in the 25 μM cisplatin condition. Error bars represent standard deviation. *N* = 1 biological replicate, *n* = 6–9 wells per condition.

As shown in Figure 3, our method allows for the identification of toxic drugs in a dose-dependent manner; here, cisplatin is a drug known for injuring and killing renal tubular cells. In some drugs, such as cisplatin, the response of the organoids to the drug can be expected to be patient specific. Indeed, cisplatin-induced nephrotoxicity is a patient-dependent phenomenon that can be linked to, among others, various genotypic variations.^49^ This patient-specific response can be observed in Figures 3B and 3D with different responses to the same concentration of cisplatin.

To confirm that organoids isolated from tumor and kidney samples are different and not originating from a similar population in both tissues, a bulk RNA sequencing (RNA-seq) was performed on one patient sample (2017-00364-RCC09-03MAY22). The transcriptomes of organoids isolated from a clear cell RCC (ccRCC) tumor and organoids isolated from contiguous non-cancerous kidney tissue were compared. The comparison showed 1,928 genes that were differentially expressed in a significant manner (fold change ≥ 2 and *p* value adjusted for false discovery rate < 0.05). To assess the stability over passages and account for mutations induced by the culture conditions, the same sequencing was performed in parallel on two samples originating from the same tissue of a different patient (2017-00364-RCC01-03OCT21) but cultivated separately for at least 3 passages. In this case, only 15 genes were differentially expressed. This shows that the tissues cultivated following this protocol retain stable phenotypes over multiple passages. Moreover, it confirms that the 1,928 differentially expressed genes observed in between the ccRCC and non-cancerous samples of patient 2017-00364-RCC09-03MAY22 were not introduced by the culture conditions but are inherent to the origin of each sample. To better visualize the similarities and dissimilarities present between the compared samples, an illustrative heatmap showing the most 500 variably expressed genes is provided in Figure S4. To confirm that our 3D structures are composed of the different cell populations expected in organoids, the RNA-seq data from the non-cancerous samples of patient 2017-00364-RCC09-03MAY22 were further studied to look for the presence of cell-specific biomarkers. The expressed genes were compared with the literature, as well as the Human Protein Atlas. Different populations of kidney cells and stem and progenitor cells were identified based on the expression of specific markers (Table S3). While this is only qualitative information on the presence of each cell subset, this confirms that the 3D structures, isolated following our method, comprise all the cell populations expected in kidney organoids.

Our selected medium and culture method allows us to preserve the undifferentiated state of some of the cells forming organoids. Flow cytometry and bulk RNA-seq data (Figure S1; Table S3) show populations of cells expressing selected stemness markers. We hypothesize that this allows us to preserve the growth of the cells for longer, as they do not undergo replicative exhaustion as fast. We also observed that patient samples grown as 2D monolayers in Renal Epithelial Cell Growth Medium 2 would typically stop growing in a matter of weeks, whereas the same sample in the same culture medium grown in 3D according to the presented protocol would not show any sign of exhaustion after more than 12 passages.

## Limitations

The main limitation of the method is tissue availability. In our experience, RCC fragments at the same weight as non-cancerous fragments contain fewer cells. Hence, to improve the chances of obtaining organoids that can expand, we recommend processing at least 60 mg of non-cancerous tissue and a minimum of 200 mg RCC tissue. Fragments any smaller than the sizes mentioned might result in a lack of growth of cells after the procedure.

Isolation can be a time-consuming procedure that can extend over normal office hours depending on the starting time and duration of the nephrectomy. To account for this issue, we tested different ways of storing the tissues before isolation. After mechanical fragmentation (steps 1–5), we tried to (1) snap freeze, (2) freeze slowly in FBS with 10% DMSO, or (3) keep the tissue at 4°C in DMEM/F12. Only the third method yielded organoids, whereas the frozen tissues appeared to contain no viable cells after thawing and isolation.

Flow cytometry analysis of the cells obtained after the isolation of samples submitted to this storage procedure did not present significantly different cell populations than the ones from the same samples isolated fresh (Figure S2). However, this storage procedure always resulted in a decrease in the ratio of cells isolated over the original mass of tissue. Furthermore, we did not attempt to keep the sample in storage for more than 48 h after surgery. Therefore, while it is possible to store the tissues for a short period of time, we recommend performing the isolation as soon as possible to maximize the success rate.

Current isolation and cultivation methods do not allow for full preservation of the complete tumor microenvironment, such as blood vessels, immune cells, and cancer-associated fibroblasts, as, over time, they get depleted in culture (Figure S1).^37^^,^^50^ It might be beneficial to isolate these cells from fresh samples by introducing an additional step of sorting if these cells are later desired to be present in the organoids. These cells could then be mixed with the organoid cells in step 43 to include them inside the 3D structures or added on top of the organoids a few days after the seeding to better recapitulate the reality of the tissue composition. Preliminary results of an ongoing project are shown in Figure S3 as an example of such a co-culture model. More complexity could also be added through the use of microfluidic organoids-on-chips to mimic immune infiltration, angiogenesis, and metastasis.^51^

Isolated samples display markers with different cell specificities, indicating the presence of multiple cell populations. However, not all markers expected to be present in these cell populations could be observed (Table S3). Thus, we suspect that although differentiation into the different kidney cell populations is happening, the differentiated cells are not fully mature. The scale of this immaturity in each cell population and its global impact on treatment response remains unknown.

## Acknowledgments

We acknowledge Prof. Laura Rubbia-Brandt for facilitating the studies and Dr. Mylene Docquier for RNA-seq analysis.

## Author contributions

Study design and plan, analysis, interpretation, and manuscript writing, J.G., V.M., and P.N.-S.; data collection, J.G., V.M., J.-C.T., and D.B.; funding, P.N.-S.; manuscript review and approval, all authors.

## Declaration of interests

The authors declare no conflicts of interest.

## Supplemental information

Document S1. Figures S1–S4 and Tables S1–S3

Document S2. Article plus supplemental information
