# Supplementary material for: Generation and maintenance of kidney and kidney cancer organoids from patient-derived material for drug development and precision oncology
Source: Mol Ther Methods Clin Dev. 2024 Nov 5;32(4):101368. doi: 10.1016/j.omtm.2024.101368 (PMC11629258; doi:10.1016/j.omtm.2024.101368)
Supplement: Document S1. Figures S1–S4 and Tables S1–S3 [file mmc1.pdf]

OMTM, Volume *32*

Supplemental information

Generation and maintenance of kidney and kidney cancer organoids from patient-derived material for drug development and precision oncology

Jakub Gubala, Valentin Mieville, Daniel Benamran, Jean-Christophe Tille, Massimo Valerio, and Patrycja Nowak-Sliwinska

SUPPLEMENTAL MATERIAL

Table S1 Role of each culture media supplements according to the literature

Supplement	Role	Ref.
hEGF	Stimulates growth of tubular cells, stimulates growth of cancer cells	[1]
hFGF2	Stimulates proliferation of variety of renal cells; allows for epithelial to mesenchymal transition of Tubular Epithelial Cells	[2]
hFGF10	Mitigates I/R induced apoptosis of Renal Cells; Maintains Histological integrity and safeguard's renal function	[3]
R-spondin 1	Stimulates the Wnt/β-catenin pathway; allows for maintenance and differentiation of stem cells	[4]
R-spondin 3		
Human noggin	Maintains undifferentiated human embryonic stem cells *in vitro*	[4]
Human Wnt-3a	Maintains proliferation of stem cells	[5]
Human PDFG-BB	Inhibits degeneration of organoids	[6]
Primocin	Antimicrobial agent	Invivogen, ant-pm-1 data sheet
StemPro® hESC	Maintains multipotential phenotype of stem cells	Gibco® Technical Sheet

Table S2 Information on isolated patient samples. Each patient sample contains information on lab ID, anonymized Patient ID, the grade and stage of the tumor, type of performed surgery, whether we obtained or not the tumor and/or healthy sample, if we managed to obtain organoids, and information on subtype of renal cancer. PRCC in LabID stands for RCC samples and PHK for kidney samples.

LabID	Patient ID	Stage / Grade	Operation	Tumor sample	Weight (mg)	ORGANOIDS FORMATION	Healthy sample	Weight (mg)	ORGANOIDS FORMATION	Comment
**PRCC1 / PHK1**	**2017-00364-RCC01-03OCT21**	pT2b	nephrectomy	**X**	**N/A**	yes	**X**	**N/A**	yes	chromophobe cell RCC
**PRCC3 / PHK3**	**2017-00364-RCC03-11OCT21**	no staging	partial nephrectomy	**-**	**-**	-	**X**	**300**	yes	inflammatory kyst
**PRCC4 / PHK4**	**2017-00364-RCC04-25OCT21**	unknown	nephrectomy	**X**	**97**	no	**X**	**152**	yes	chRCC
**PRCC5 / PHK5**	**2017-00364-RCC05-07DEC21**	ISUP 2 pT3a	nephrectomy	**X**	**934**	yes	**X**	**499**	yes	ccRCC
**PRCC6 / PHK6**	**2017-00364-RCC06-14DEC21**	ISUP 2 pT1a	partial nephrectomy	**X**	**3513**	yes	**X**	**230**	yes	Papillary RCC
**PRCC8 / PHK8**	**2017-00364-RCC08-03MAR22**	ISUP 3	partial nephrectomy	**X**	**230**	no	**-**	**-**	-	ccRCC
**PRCC9 / PHK9**	**2017-00364-RCC09-03MAY22**	ISUP 3	nephrectomy	**X**	**273**	yes	**X**	**167**	yes	ccRCC
**PRCC13 / PHK13**	**2017-00364-RCC13-27JUN23**	ISUP 2 pT1a	tumorectomy	**X**	**65**	yes	**-**	**-**	-	ccRCC
**PRCC14 / PHK14**	**2017-00364-RCC14-22AUG23**	no staging	tumorectomy	**X**	**134**	yes	**-**	**-**	-	Oncocytoma
**PRCC15 / PHK15**	**2017-00364-RCC15-26SEP23**	ISUP 2 pT1a	tumorectomy	**X**	**198**	no	**X**	**42**	no	ccRCC
**PRCC16 / PHK16**	**2017-00364-RCC16-26SEP23**	pT1a	tumorectomy	**X**	**39**	no	**X**	**83**	no	chromophobe cell RCC
**PRCC17 / PHK17**	**2017-00364-RCC17-21NOV23**	ISUP2 pT1b	tumorectomy	**X**	**62**	no	**-**	**-**	-	ccRCC and scar tissue-like part
**PRCC18 / PHK18**	**2017-00364-RCC18-22JAN24**	ISUP 2 pT1a	tumorectomy	**X**	**1491**	yes	**X**	**185**	yes	papillary RCC
**PRCC19 / PHK19**	**2017-00364-RCC19-23JAN24**	ISUP 3 pT1a	tumorectomy	**X**	**609**	yes	**X**	**N/A**	yes	papillary RCC
**PRCC20 / PHK20**	**2017-00364-RCC20-12FEB24**	ISUP 3 pT3a pN1	partial nephrectomy	**X**	**648**	yes	**X**	**129**	yes	ccRCC
**PRCC21 / PHK21**	**2017-00364-RCC21-20FEB24**	pT1a	tumorectomy	**X**	**391**	yes	**X**	**69**	yes	chromophobe cell RCC
**PRCC22 / PHK22**	**2017-00364-RCC22-11MAR24**	ISUP 3 pT2b pN0	nephrectomy	**X**	**1435**	yes	**X**	**145**	yes	ccRCC
**PRCC23 / PHK23**	**2017-00364-RCC23-12MAR24**	pT1a	tumorectomy	**X**	**350**	yes	**X**	**57**	yes	chromophobe cell RCC
**PRCC24 / PHK24**	**2017-00364-RCC24-18APR24**	ISUP 3 pT1b	partial nephrectomy	**X**	**258**	no	**X**	**101**	no	ccRCC
**PRCC25 / PHK25**	**2017-00364-RCC25-07MAY24**	ISUP 2 pT3a N0	nephrectomy	**X**	**964**	yes	**X**	**537**	yes	ccRCC
**PRCC26 / PHK26**	**2017-00364-RCC26-14MAY24**	ISUP 3 pT1a	tumorectomy	**X**	**252**	no	**X**	**165**	yes	ccRCC
**PRCC27 / PHK27**	**2017-00364-RCC27-28MAY24**	ISUP 2 pT1b	tumorectomy	**X**	**589**	yes	**X**	**243**	yes	ccRCC
**PRCC28 / PHK28**	**2017-00364-RCC28-18JUN24**	pT1a	tumorectomy	**X**	**207**	yes	**X**	**49**	yes	chRCC

Table S3 : Non-exhaustive list of gene expressed or not in the non-cancerous sample of patient 2017-00364-RCC09-03MAY22 and their attribution to different cell populations based on the literature.

Gene	Individual replicas count			Average count	Origin	Ref
POU5F1	123	96	89	103	Stem / progenitor cells	[7]
KLF4	3140	1127	2009	2092		[7]
SOX9	18288	12281	18660	16410		[8]
CD44	22469	17626	24406	21500		[9]
ALCAM	6203	5551	9748	7167		[10]
CD24	6916	5181	7591	6563		[9]
PAX2	24	25	29	26		[9]
PAX8	148	125	145	139		[8]
CITED1	119	189	217	175		[11]
NOTCH2	2550	2553	3751	2951		[12]
SIX2	0	0	0	0		[8]
PODXL	3563	3236	5406	4068	Podocytes	[13]
WT1	268	403	705	459		[8,12,14]
CD2AP	3498	1995	4259	3251		[14]
NPHS1	2	0	0	1		[12,14]
ABCC1	1747	2506	3721	2658	Proximal tubule / loop of Henle cells	[8]
ABCC3	2417	3305	4290	3337		[8]
ABCC4	4445	6198	9509	6717		[8]
CDH16	154	18	18	63		[13]
ATP11A	1296	1006	1595	1299		[12]
SLC26A6	1283	702	759	915		[12]
PPARA	1922	1086	1635	1548		[12]
GLUD1	14126	10956	14898	13327		[12]
CALD1	5786	4775	8062	6208		[12]

CLCN5	1600	1447	2238	1762		[15]
LRP2	0	0	0	0		[12,14]
PROM2	3820	2924	3691	3478		[16]
UMOD	0	0	0	0		[12]
PCBD1	3411	5170	6340	4974	Distal tubule cells	[8]
CALB1	15872	11001	16632	14502		[12]
ATP1A1	62438	35466	45395	47766		[12]
SLC12A3	0	0	0	0		[12]
CDH1	11293	6979	10401	9558	Collecting duct cells	[8]
AQP3	3086	1410	1605	2034		[8]
AQP2	0	2	3	2		[12,14]

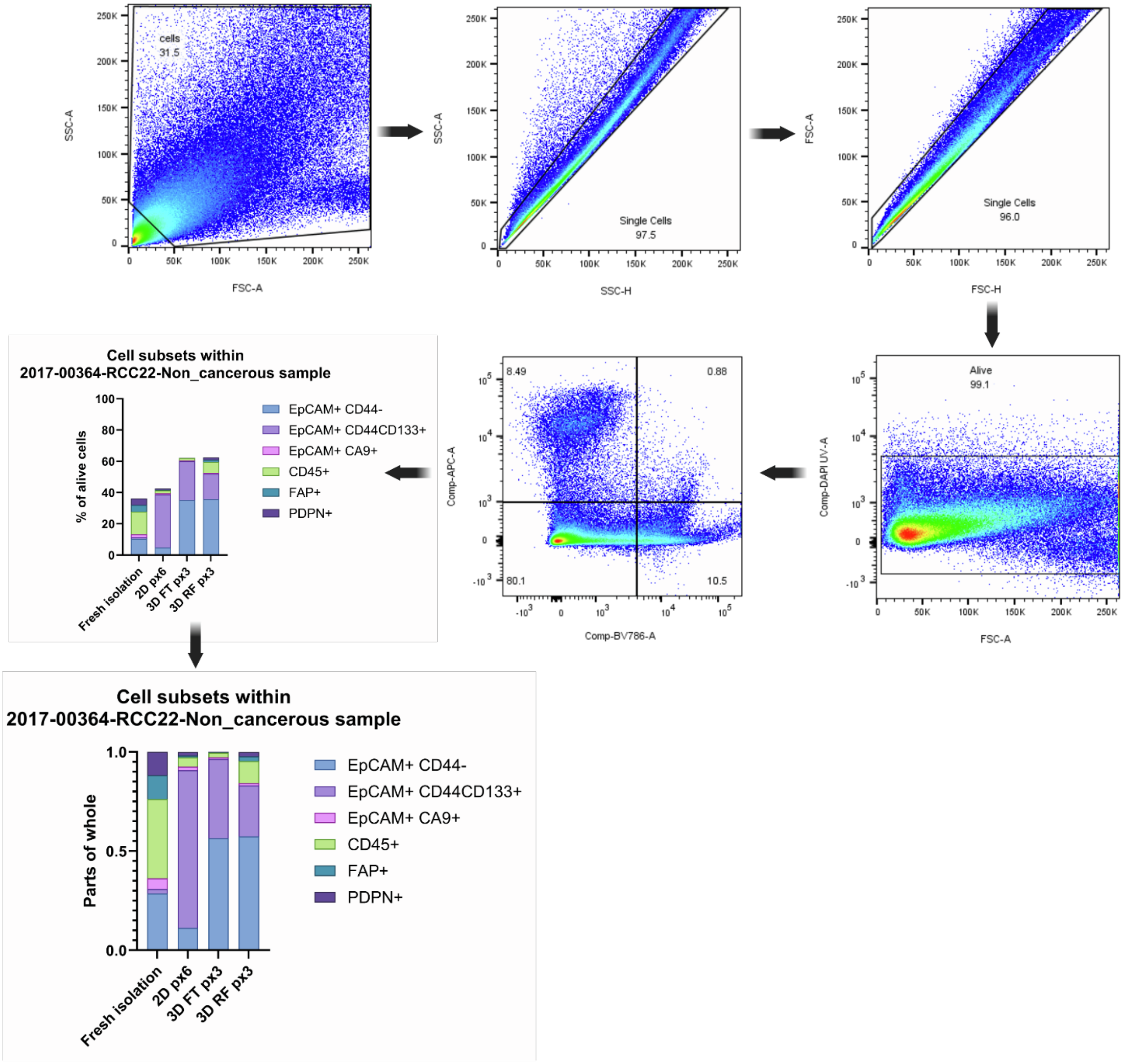

Figure S1 Flow cytometry analysis of cellular composition of freshly digested (2017-00364-RCC22-11MAR24) non-cancerous sample and of organoids / cells kept in 2D and 3D accordingly. 2D cells were kept in culture for 6 splits and 3D for 3 splits. The composition of both Flow Through (FT) fraction and Retained Fraction (RF) were assessed. The composition of all samples was normalized to all cells detected with selected markers. Parts of debris might appear in flow cytometry readout as a live cell, hence normalization to all detected cells allows to compare the compositions in between the samples without taking into account the debris. In both 2D and 3D cultures after a certain number of splits, we noticed a depletion of immune cells (CD45+) as well as of cells expressing Cancer Associated Fibroblasts markers

(CAF+ and PDPN+). We also observed higher fraction of cells expressing stemness/progenitor markers (CD44+CD133+).

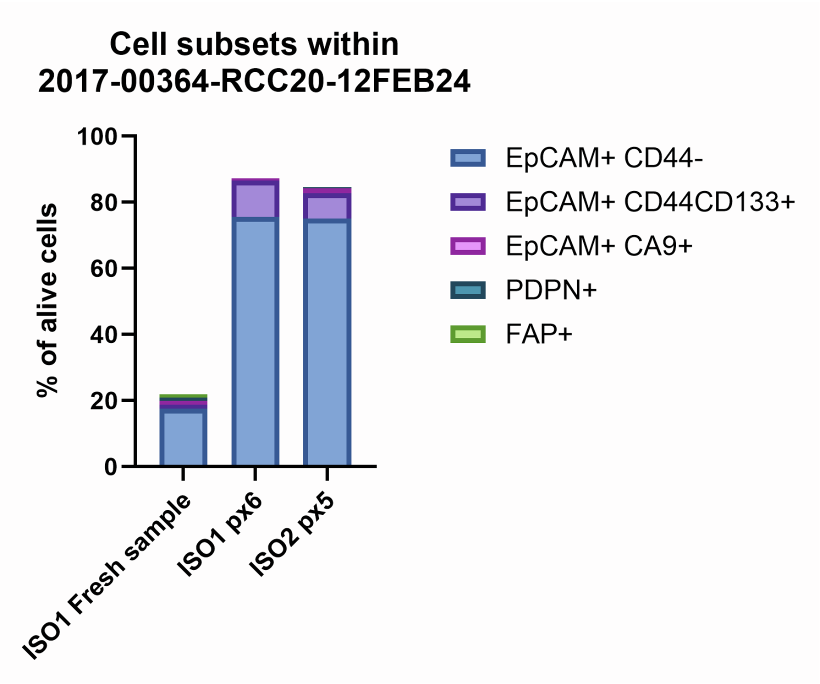

Figure S2 Flow cytometry analysis of the cellular composition of freshly digested (2017-00364-RCC20-12FEB24) non-cancerous samples and of organoids kept in 3D accordingly. The same tissue was divided into equal parts and isolated after storing in different conditions. ISO1 is a sample that was isolated directly upon receiving, and ISO2 is a sample that was isolated after storage for 48h in DMEM/F12 + Primocin at 4°C. Despite similar size of the tissues when attempting isolation, yield of cells was significantly lower when compared to fresh isolation. Despite that fact, it did not affect the growth of the organoids, or their composition after several splits.

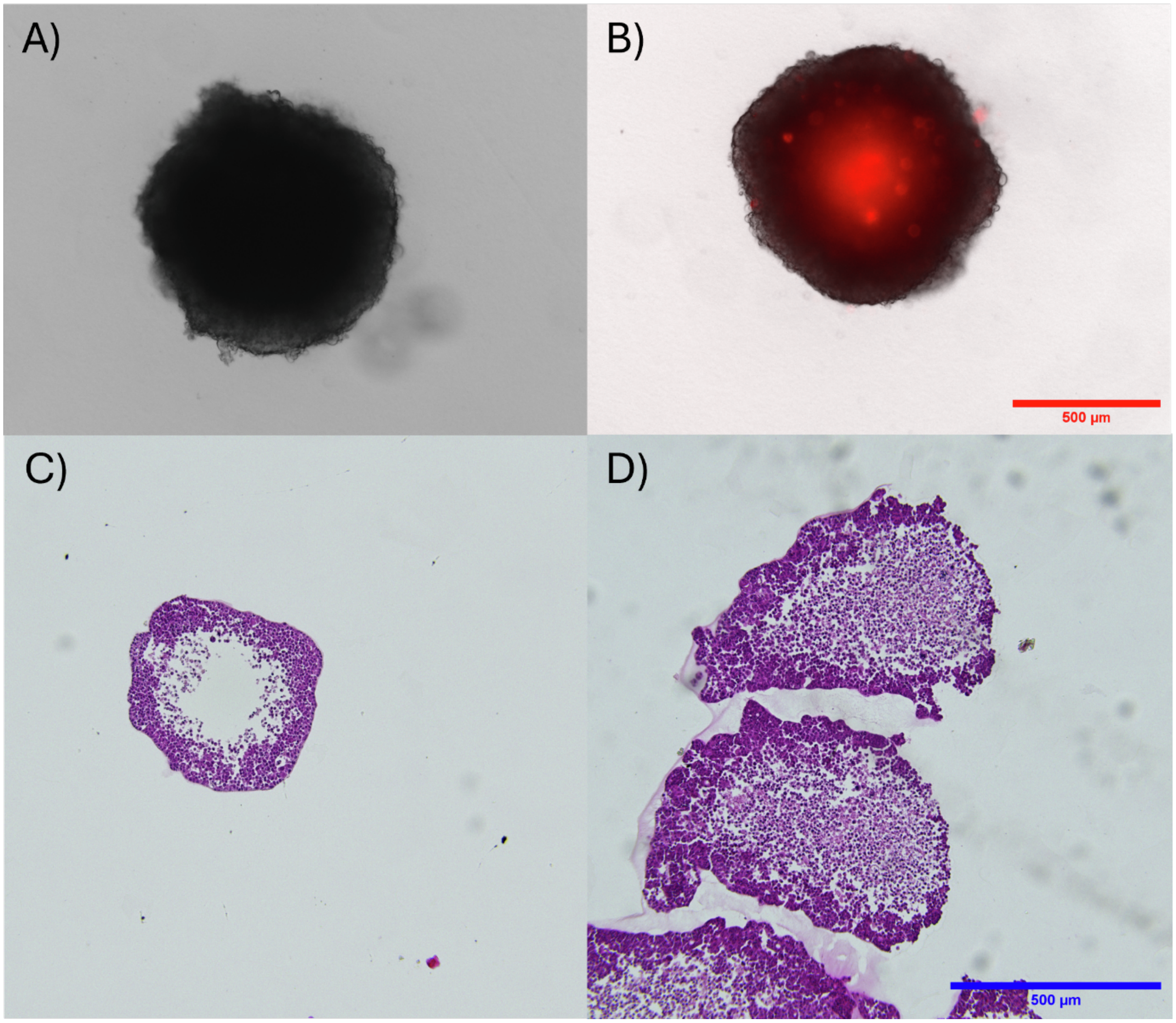

Figure S3 Organoids derived from the tumor of 2017-00364-RCC09-03MAY22. Images A) and C) represent mono-cultured single organoids, 14 days into culture, whereas B) and D) represent organoids co-cultured with Normal Human Dermal Fibroblasts, initially added as 10% of all cells. B) Fibroblasts were stained with red live cell tracker and cultured together with cancer cells for 14 days. Images C) and D) represent H&E staining of sections of single organoids. Several single organoids were pulled together, fixed with 4% PFA in PBS, dehydrated, embedded in paraffin and cut into 5µm thick sections.

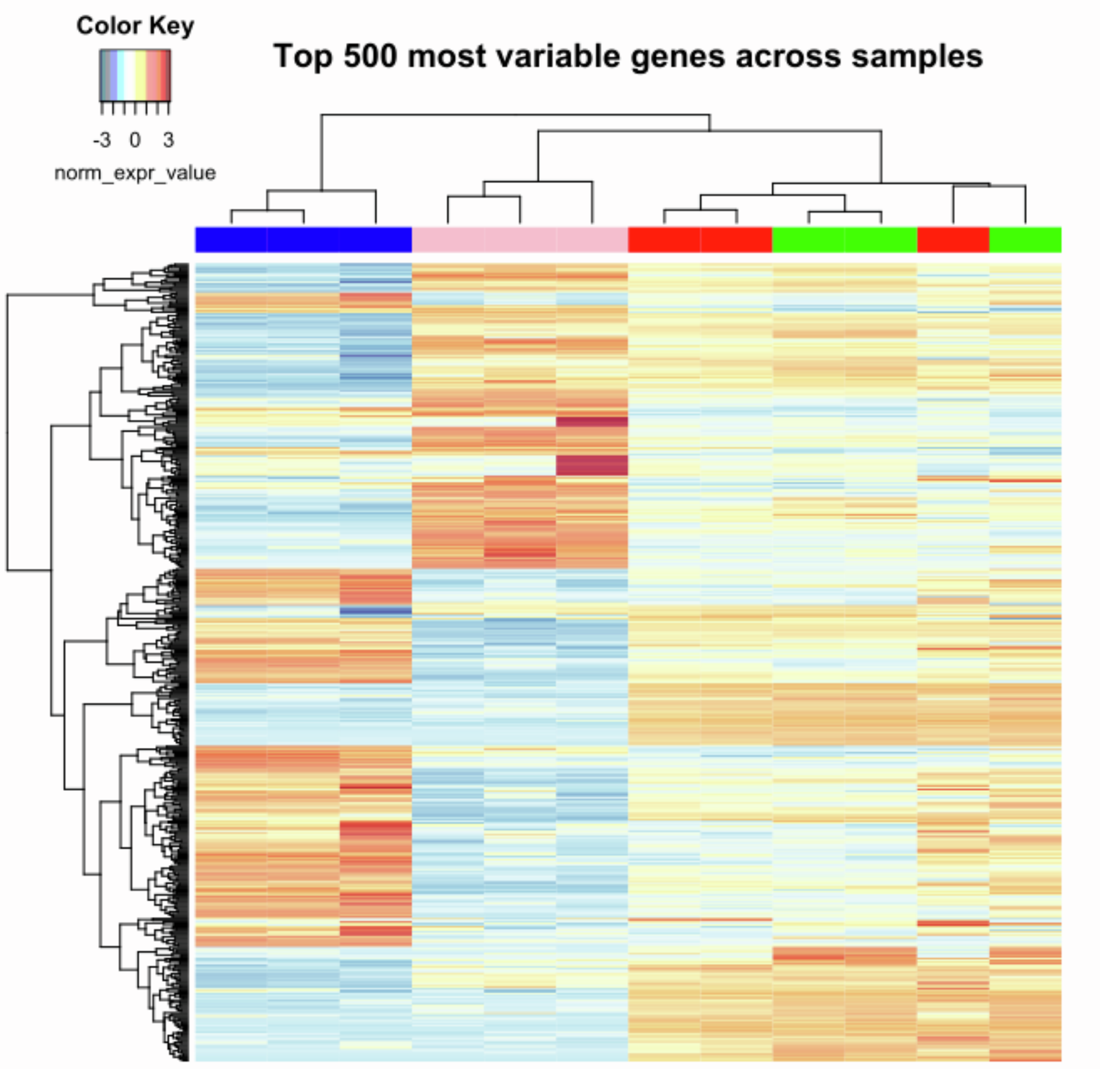

Figure S4 Bulk RNA sequencing results of four organoids samples displayed as a matrix of Euclidean distances from the log2 normalized counts for the 500 most variable genes represented by a heatmap. Each row represents the expression of a different gene. Blue color means the expression is downregulated and red color means it is upregulated. Colors on top of the columns represent different samples. Pink and blue are ccRCC and kidney organoids from the patient 2017-00364-RCC09-03MAY22. Red and green are two samples that were cultivated separately after their isolation from the patient 2017-00364-RCC01-03OCT21. Each column is a biological replicate.

1. Rauth, S., Karmakar, S., Batra, S.K., and Ponnusamy, M.P. (2021). Recent advances in organoid development and applications in disease modeling. Biochim Biophys Acta Rev Cancer *1875*, 188527. 10.1016/j.bbcan.2021.188527.
2. Strutz, F. (2009). The role of FGF-2 in renal fibrogenesis. Front Biosci (Schol Ed) *1*, 125-131. 10.2741/S12.
3. Tan, X., Yu, L., Yang, R., Tao, Q., Xiang, L., Xiao, J., and Zhang, J.S. (2020). Fibroblast Growth Factor 10 Attenuates Renal Damage by Regulating Endoplasmic Reticulum Stress After Ischemia-Reperfusion Injury. Front Pharmacol *11*, 39. 10.3389/fphar.2020.00039.
4. Urbischek, M., Rannikmae, H., Foets, T., Ravn, K., Hyvönen, M., and de la Roche, M. (2019). Organoid culture media formulated with growth factors of defined cellular activity. Sci Rep-Uk *9*. ARTN 6193 10.1038/s41598-019-42604-0.
5. Sato, T., Stange, D.E., Ferrante, M., Vries, R.G., Van Es, J.H., Van den Brink, S., Van Houdt, W.J., Pronk, A., Van Gorp, J., Siersema, P.D., et al. (2011). Long-term expansion of epithelial organoids from human colon, adenoma, adenocarcinoma, and Barrett's epithelium. Gastroenterology *141*, 1762-1772. 10.1053/j.gastro.2011.07.050.
6. Zhang, W., Gong, Y., Zheng, X., Qiu, J., Jiang, T., Chen, L., Lu, F., Wu, X., Cheng, F., and Hong, Z. (2021). Platelet-Derived Growth Factor-BB Inhibits Intervertebral Disc Degeneration via Suppressing Pyroptosis and Activating the MAPK Signaling Pathway. Front Pharmacol *12*, 799130. 10.3389/fphar.2021.799130.
7. Takahashi, K., and Yamanaka, S. (2006). Induction of Pluripotent Stem Cells from Mouse Embryonic and Adult Fibroblast Cultures by Defined Factors. Cell *126*, 663-676. 10.1016/j.cell.2006.07.024.
8. Schutgens, F., Rookmaaker, M.B., Margaritis, T., Rios, A., Ammerlaan, C., Jansen, J., Gijzen, L., Vormann, M., Vonk, A., Viveen, M., et al. (2019). Tubuloids derived from human adult kidney and urine for personalized disease modeling. Nature Biotechnology *37*, 303-313. 10.1038/s41587-019-0048-8.
9. Huang, J., Kong, Y., Xie, C., and Zhou, L. (2021). Stem/progenitor cell in kidney: characteristics, homing, coordination, and maintenance. Stem Cell Research & Therapy *12*, 197. 10.1186/s13287-021-02266-0.
10. Halfon, S., Abramov, N., Grinblat, B., and Ginis, I. (2011). Markers distinguishing mesenchymal stem cells from fibroblasts are downregulated with passaging. Stem Cells Dev. *20*, 53-66. 10.1089/scd.2010.0040.
11. Boyle, S., Shioda, T., Perantoni, A.O., and de Caestecker, M. (2007). Cited1 and Cited2 are differentially expressed in the developing kidney but are not required for nephrogenesis. Dev Dyn *236*, 2321-2330. 10.1002/dvdy.21242.
12. Balzer, M.S., Rohacs, T., and Susztak, K. (2022). How Many Cell Types Are in the Kidney and What Do They Do? Annu Rev Physiol *84*, 507-531. 10.1146/annurev-physiol-052521-121841.
13. Hiratsuka, K., Monkawa, T., Akiyama, T., Nakatake, Y., Oda, M., Goparaju, S.K., Kimura, H., Chikazawa-Nohtomi, N., Sato, S., Ishiguro, K., et al. (2019). Induction of human pluripotent stem cells into kidney tissues by synthetic mRNAs encoding transcription factors. Sci Rep *9*, 913. 10.1038/s41598-018-37485-8.
14. Agarwal, S., Sudhini, Y.R., Polat, O.K., Reiser, J., and Altintas, M.M. (2021). Renal cell markers: lighthouses for managing renal diseases. Am J Physiol Renal Physiol *321*, F715-F739. 10.1152/ajprenal.00182.2021.
15. Gianesello, L., Del Prete, D., Ceol, M., Priante, G., Calò, L.A., and Anglani, F. (2020). From protein uptake to Dent disease: An overview of the CLCN5 gene. Gene *747*, 144662. 10.1016/j.gene.2020.144662.

16. Jászai, J., Farkas, L.M., Fargeas, C.A., Janich, P., Haase, M., Huttner, W.B., and Corbeil, D. (2010). Prominin-2 is a novel marker of distal tubules and collecting ducts of the human and murine kidney. Histochem Cell Biol *133*, 527-539. 10.1007/s00418-010-0690-1.
